# Uncontrolled Hypertension and Its Determinants in Patients with Concomitant Type 2 Diabetes Mellitus (T2DM) in Rural South Africa

**DOI:** 10.1371/journal.pone.0150033

**Published:** 2016-03-01

**Authors:** Oladele Vincent Adeniyi, Parimalaranie Yogeswaran, Benjamin Longo-Mbenza, Daniel Ter Goon

**Affiliations:** 1 Department of family medicine, Walter Sisulu University, East London, South Africa; 2 Department of family medicine, Walter Sisulu University, Mthatha, South Africa; 3 University of Fort Hare, East London, South Africa; University of Perugia, ITALY

## Abstract

**Background:**

Paucity of data on the prevalence, treatment and control of hypertension in individuals living with type 2 diabetes mellitus (T2DM) in the rural communities of South Africa may undermine efforts to reduce the morbidity and mortality associated with cardiovascular diseases. This study examines the socio-demographic and clinical determinants of uncontrolled hypertension among individuals living with T2DM in the rural communities of Mthatha, South Africa.

**Methods:**

This cross-sectional study involved a serially selected sample of 265 individuals living with T2DM and hypertension at Mthatha General Hospital, Mthatha. Uncontrolled hypertension was defined as systolic blood pressure greater than or equal to 140mmHg and diastolic blood pressure greater than or equal to 90mmHg in accordance with the Eight Joint National Committee Report (JNC 8) (2014). We performed univariate and multivariate logistic regression analyses to identify the significant determinants of uncontrolled hypertension.

**Results:**

Of the total participants (n = 265), the prevalence of uncontrolled hypertension was 75.5% (n = 200). In univariate analysis of all participants, male gender (p = 0.029), age≥65 years (p = 0.016), unemployed status (p<0.0001), excessive alcohol intake (p = 0.005) and consumption of western-type diet (p<0.0001) were positively associated with uncontrolled hypertension. In multivariate logistic regression (LR method) analysis, unemployed status (p<0.0001), excessive alcohol intake (p = 0.007) and consumption of western-type diet (p<0.0001) were independently and significantly associated with uncontrolled hypertension. There is significant association between increasing number and classes of anti-hypertensive drugs and uncontrolled hypertension (p = 0.05 and 0.02, respectively).

**Conclusion:**

Prevalence of uncontrolled hypertension was high in individuals with concomitant hypertension and T2DM in the study population. Male sex, aging, clinic inertia, unemployed status and nutritional transitions are the most important determinants of uncontrolled hypertension in T2DM in Mthatha, South Africa. Treatment to blood pressure targets, though feasible in our setting, would require concerted efforts by addressing these determinants and clinic inertia.

## Introduction

Globally, hypertension is the leading cause of cardiovascular diseases (CVD) and deaths [1], and accounts for about 7.5 million deaths per year [2]. Hypertension tends to occur in clusters with other cardiovascular diseases such as type 2 diabetes (T2DM) and dyslipidaemia. Between 20–60% of individuals with T2DM will have concomitant hypertension, and the co-morbidity varies with age, ethnicity, and body mass index [1]. Hypertension doubles the risk of all-cause mortality and stroke, triples the risk of coronary artery disease and accelerates the progression of diabetic nephropathy, retinopathy, and neuropathy [3]. Like most cardiovascular diseases, the natural course of hypertension can be modified with the use of effective and inexpensive medications [1].

The benefits of achieving blood pressure control in individuals with co-morbid T2DM have been well documented [4–7]. Many randomized controlled trials have shown unequivocally that treatment of hypertension reduces the risk of stroke, coronary heart disease, congestive heart failure and mortality [8,9]. Also, the Seven Countries Study demonstrated a 15% risk reduction for cardiovascular deaths [10]. It is imperative that lowering of blood pressure to treatment targets is therefore, a priority in individuals with T2DM to prevent major cardiovascular and renal events [1].

There seems to be inconsistencies in the recommendations by different authorities on the blood pressure (BP) treatment targets in individuals with T2DM. The Society of Endocrinology, Metabolism and Diabetes of South Africa (SEMDSA) sets BP target of less than 140/80mmHg in patients with T2DM [11]. This recommendation was supported by the American Diabetes Association [12]. However, BP target of less than 140/90mmHg was recommended by European hypertension and Latin American hypertension guideline for individuals with T2DM and the rest of the population [1,13]. Also, the recently published Eight Report of the Joint National Committee (JNC 8) has confirmed the BP less than 140/90mmHg as the desired treatment target for people living with T2DM [14].

Many studies have reported sub-optimal BP control among hypertensive individuals worldwide [15–19]; 44.6% among elderly hypertensive patients in China [15], 29.2% was found in 1999–2000 and 36.8% in 2003–2004 in US population [19]. Similar trends of poor control of hypertension have been documented across African countries namely, Ethiopia (42.2%), Mozambique (39.9%), Algeria (37.5%), Ghana (6.2%) and South Africa (31%) [17].

Reasons for poor control of hypertension are complex and varied across regions. Patients’ non-adherence, physician inertia to treat to targets, and health system factors; lack of anti-hypertensive medications and long travels to health facilities have been reported in many African countries [17]. Blood pressure control appears to be associated with socio-demographic factors; younger patients and women were positively associated with better blood pressure control [17]. The associated factors of control of hypertension among adult rural residents of Mthatha, Eastern Cape, South Africa and especially those with concomitant T2DM remain unexplored.

The high proportion of overweight and obese adults living with T2DM coupled with the changing patterns of diet in the rural communities of Mthatha reported by Adeniyi et al. [20] may impact on the control of hypertension. Also, dyslipidaemia though, plays significant role in the pathogenesis of hypertension in individuals with concomitant T2DM, its association with the control of blood pressure have not been investigated in the rural communities in South Africa. There is still paucity of data on the prevalence, treatment and BP control among individuals with concomitant hypertension and T2DM in the rural communities of South Africa. Epidemiologic data on the factors associated with control of hypertension among high risk groups especially T2DM will provide useful insights to the implementation of evidence-based guideline and thus, contribute towards the global and country efforts towards addressing the scourge of non-communicable diseases. This study examines the prevalence of uncontrolled hypertension using JNC 8 blood pressure treatment target criteria and the associated factors among individuals with co-morbid T2DM in the predominant rural residents of Mthatha, South Africa.

## Methods

### Study Recruitment

This study presents analysis of data on uncontrolled hypertension among 360 adults (30 years and above) living with T2DM at Mthatha General Hospital, Mthatha, South Africa. The detailed methods of this cross-sectional study have previously been described elsewhere [20].

Briefly, participants with history of treatment for T2DM for at least one year and attending follow up care at Mthatha General Hospital, Eastern Cape, South Africa from July to November, 2013 were recruited for the study. This hospital provides level one care (district healthcare service) and supervision for about 15 community health centres and clinics from the rural communities surrounding Mthatha. We excluded 33 participants with incomplete medical data from the study. The current analysis included only participants with concomitant hypertension and T2DM (n = 265) out of the 327 participants (81%).

### Ethical approval

The Walter Sisulu University Ethics Committee granted approval for the study protocol, consent form and participants’ information sheet (Protocol number: 031/2013 dated on 9^th^ October, 2013). The Eastern Cape Department of Health as well as the Mthatha General Hospital management gave permission to conduct the study. Participants were provided information sheet written in both English and IsiXhosa languages, detailing the purpose, process of the research, rights of the participants, and contact details of whom to contact about the research prior to obtaining written informed consent. Each participant signed informed consent indicating voluntary participation in the study. Participants’ rights to privacy and confidentiality were respected throughout the study proceedings in accordance with Helsinki Declaration.

### Data collection

Data were collected through interview and review of medical records. Participant’s age, sex, level of education, marital status, type of residence, employment status, personal monthly income, dietary contents, smoking status, soft drink consumption, and physical activity were obtained through interview. Duration of diabetes, types of medications, and other co-morbid conditions such as hypertension, stroke, and heart failure were extracted from the medical records.

Level of education was determined by the maximum grade level attained in school. They were categorized into no formal education, primary (grades 1–6), secondary education (grade 7–12) or tertiary (post-secondary) education. Participants were further classified as unemployed if they had no occupation in either formal or informal sector. Monthly income was categorized into low (less than 1200 South African rand) and high (more than 1200 South African rand).

Smoking status was determined by the frequency of cigarette smoking and categories as current smoker (smoking at least one cigarette within the past month), former smoker (having quit smoking more than one month prior to the study). Alcohol consumption was categorized as excessive drinkers (≥3 units/day for men and 2 units/day for women), or not (≤2 units/day for men and ≤ 1 unit/day for women).

Physical activity was categorized as either inactive or active based on the exercise being capable to increase the heart rate and respiratory rate such as gardening and reported viewing of television for less than eight hours daily. Participants reported on the weekly consumption of fried and sweet food, processed food and red meat, refined grains and high-fat dairy products and added salts (Western-type diet).

### Anthropometric measurements

The height of each participant was measured without shoes using a mounted stadiometer to the nearest 0.1cm. The participants were weighed without heavy clothing to the nearest 0.1kg using a digital scale (Tanita-HD 309, Creative Health Products, MI, USA). Body mass index was then calculated as the ratio of weight in kilogramme (kg) to height in metre squared (m^2^). Participants were defined as obese in accordance with world health organization criteria [21]; if the BMI was greater than or equal to 30.0 kg/m^2^. They were then categorized into class 1 (30.0–34.9 kg/m^2^), class 2 (35.0–39.9 kg/m^2^) and class 3 (≥40 kg/m^2^). The rest of the participants were classified as overweight (25.0–29.9 kg/m^2^), normal (18.5–24.9 kg/m^2^) and underweight (<18.5 kg/m^2^).

### Blood pressure measurement

Blood pressure was measured using a Microlife BP A100 Plus model after patients had rested for at least five minutes with arm at the level of the heart and the feet together. This instrument is equipped with a single and repeated measure function, which measures blood pressure three times and displays a calculated average value. Appropriate cuff sizes were used depending on the size of participant’s arm. Each participant answered questions on the awareness and treatment of hypertension, while diagnosis of hypertension and types of medications were extracted from the medical records. Uncontrolled hypertension was defined in accordance with the BP treatment targets recommended by the Eight Joint National Committee Criteria (2014) of systolic blood pressure greater than or equal to 140mmHg and diastolic blood pressure of greater than or equal to 90 mmHg [14]

### Laboratory assays

Fasting total cholesterol, triglycerides, high-density lipoprotein cholesterol (HDL-C), and low-density lipoprotein cholesterol (LDL-C) were measured from venous blood samples. Following overnight fast (> 8 hours without food), venous blood sample was drawn by the attending clinician in a private consulting room from the brachial vein into gel separator tubes. Blood samples were kept at room temperature for 2 hours before centrifuging for 5 minutes at 3000 rpm to separate serum from cellular elements. Serum was collected into microeppendorff tubes and stored at -80°C until processed. The fully automated Cobas^®^ C501/502 (Roche) system was used for determining serum lipid profiles. Serum creatinine level was also assayed using standard protocols.

### Statistical Analysis

We performed statistical analyses using the Statistical Package for Social Science (SPSS) version 21 for Windows (SPPSS Inc., Chicago, IL, USA). Data were expressed as mean values ± standard deviations (SD) for continuous variables. Frequency and proportions were reported for categorical variables. Percentages were compared using Chi-square test. Student’s t-test was used to compare means between groups. Univariate odd ratios (ORs) using Maentel-Haenszel and multivariate ORs and their 95% confidence intervals (95% CI) using logistic regression analysis were applied to identify the significant correlates of uncontrolled hypertension. We performed logistic regression analysis using JNC 8 criteria and adjusted for ages, monthly income, duration of T2DM and HDL-C. The p-value of < 0.05 was considered statistically significant.

## Results

Of the 265 participants with concomitant hypertension and T2DM and were all on anti-hypertensive treatment, 200 had uncontrolled hypertension (75.5%). Table 1 shows significant associations of male sex, unemployment, excessive alcohol intake, adherence to western-type diet and anti-hypertensive medications with prevalent uncontrolled hypertension.

**Table 1:** Univariate significant determinants of uncontrolled hypertension (HPT).

Variables of interest	Presence of Uncontrolled HPT		
	n (%)	OR (95%CI)	P-value
**Gender**			
Males	63/75 (84)	2 (1.02–4.1)	0.029
Females	137/190 (72.1)	1	
**Age groups**			
≥ 65 years	87/105 (82.9)	2 (1.1–3.7)	0.016
<65 years	113/160 (70.8)	1	
**Employment status**			
Unemployed	185/225 (82.2)	7.7 (3.7–15.9)	<0.0001
Employed	15/40 (37.5)	1	
**Excessive alcohol intake**			
Yes	82/94 (87)	3(1.1–7.8)	0.005
No	116/227 (73.1)	1	
**Adherence to anti-HPT medications**			
Yes	34/38 (89.5)	3 (1.1–9.2)	0.019
No	116/227 (73.1)	1	
**Adherence to Western-type diet**			
Yes	125/137 (91.2)	7.4 (3.7–14.7)	<0.0001
No	75/128 (58.6)	1	

HPT = hypertension, OR = odd ratio

However, other variables were not significantly associated with uncontrolled hypertension (p>0.05). Significant associations were observed across increasing number and classes of anti-hypertensive drugs (clinic inertia) (Table 2).

**Table 2:** Distribution of uncontrolled hypertension by antihypertensive drugs.

Variables	Uncontrolled Hypertension	P-value
**Number of drugs**	43/56 (76.8)	
1	12/12(100)	
2	137/189 (72.5)	0.005
3	8/8 (100)	
≥ 4		
**Classes of drugs**		
Thiazide alone	40/53 (75.5)	
Thiazide + ACEIs + Ca-Blockers	134/186 (72)	
B-blockers	9/9 (100)	0.022
Others	17/17 (100)	

ACEIs-angiotensin-converting enzyme inhibitors, Ca-Blockers-Calcium channel blockers

After adjusting for sex and age groups using logistic regression model analysis, the most significant independent determinants of prevalent uncontrolled hypertension were unemployment, current excessive drinker of alcohol and adherence to western-type diet (Table 3).

**Table 3:** Independent determinants of prevalent uncontrolled hypertension using logistic regression model analysis.

Independent variables	B	SE	Wald	OR (95%CI)	p-value
**Employment status**					
Unemployed	1.94	0.423	20.694	6.9 (3–15.7)	<0.0001
Employed				Reference 1	
**Western-type diet**					
Yes	1.828	0.371	24.294	6.2 (3–12.9)	0.0001
No				Reference 1	
**Excessive alcohol intake**					
Yes	1.058	0.390	7.344	2.9 (1.3–6.2)	<0.007
No				Reference 1	
Constant	-1.451	0.415	12.199		<0.0001

In considering all T2DM participants, three distinct groups emerged: absence of hypertension (n = 62), controlled hypertension (n = 65) and uncontrolled hypertension (n = 200). Table 4 highlights significant variations of mean values of ages, monthly income, BMI, HDL-C, LDL-C, LDL/HDL ratio, T2DM duration and creatinine levels across groups of hypertension.

**Table 4:** Comparisons of means of some significant determinants of uncontrolled hypertension in T2DM patients.

Variables of interest	Absence of HPT	Controlled HPT	Uncontrolled HPT	p-value
Age (Years)	52.2±12.1	56.6±12.7	61.3±11.8	<0.001
Monthly Income (Rand)	1468.9±269.7	1693.7±249.5	2847±349.7	0.02
BMI (kg/m^2^)	29.1±4.9	32.5±5.8	32.7±5.6	<0.0001
HbA1c (%)	10.2±8.4	10.6±4.1	10.3±3.4	0.837
HDL-C (mmol/L)	1.4±1.4	1.1±0.3	1.1±0.4	0.004
LDL-C (mmol/L)	4.2±1.5	3.8±1.8	2.3±1.4	<0.0001
LDL/HDL Ratio	3.8±1.1	3.7±1.3	2.8±1.1	<0.0001
T2DM Duration (Years)	4.2±3.6	6.4±5.5	7.2±6.4	0.003
Creatinine (umol/L)	86.7±45.1	125.5±88.1	120.3±79.3	0.009

BMI = body mass index, HPT = hypertension, T2DM = type 2 diabetes mellitus, LDL-C = low density lipoprotein cholesterol, HDL-C = high density lipoprotein cholesterol, HbA1c = glycated haemoglobin

**Fig 1:**
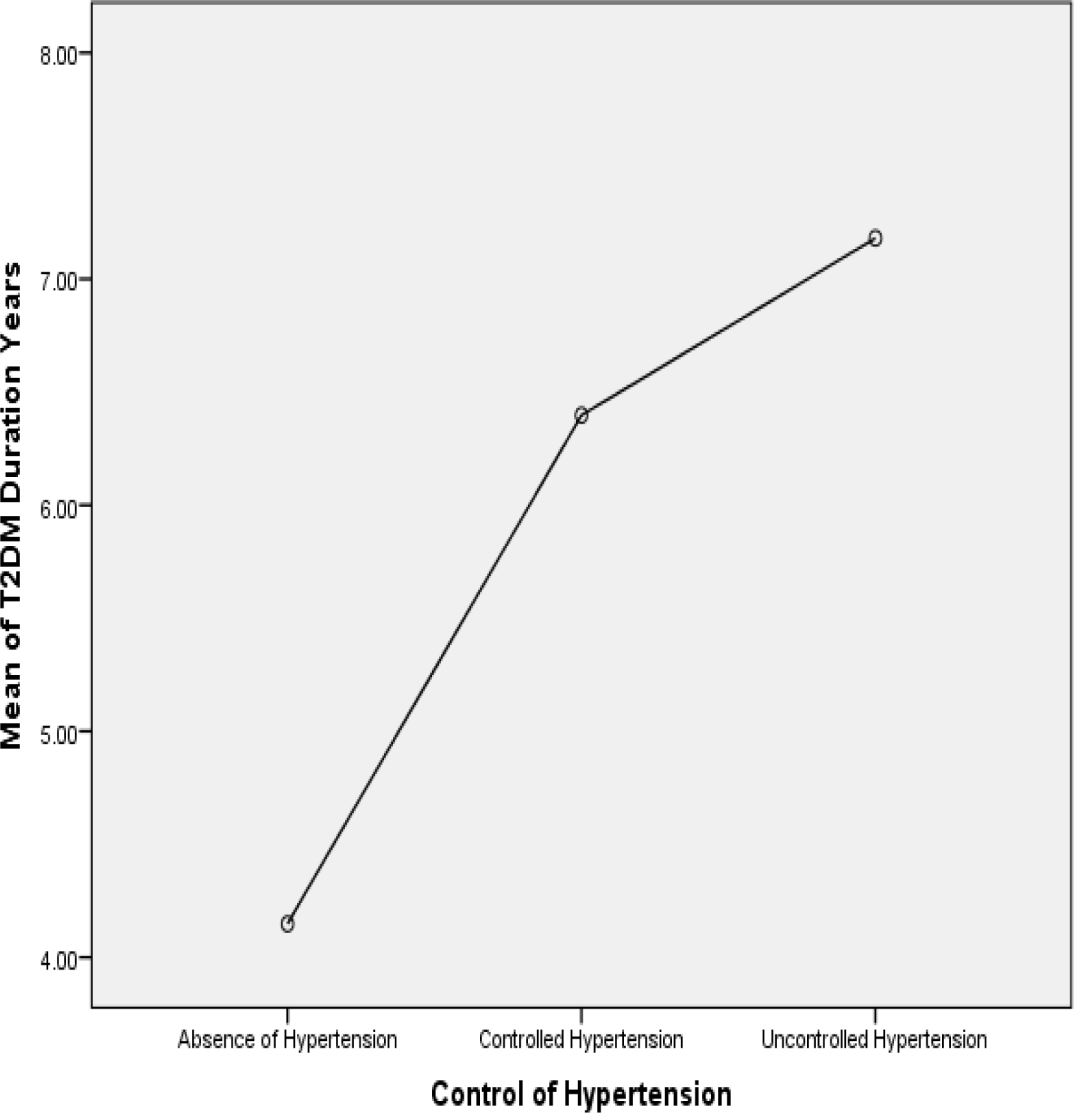
Association of T2DM duration and control of hypertension.

There is a positive linear association between the mean duration of T2DM and the hypertension groups (Fig 1).

In multivariate analysis, after adjusting for age of participants, monthly income, duration of T2DM and HDL-C, the analysis for Fisher’s linear discriminant functions at separating the study groups, only LDL-C (Tolerance = 0.999, Wiks’ Lambda = 0.948) and BMI (Tolerance = 0.965, Wiks’ Lambda = 0.923) were significant.

## Discussion

The study established the high level of co-morbidities among the rural residents of Mthatha, South Africa. The high prevalence of hypertension (81%) among people living with T2DM is not surprising. This has been alluded to in many studies worldwide [1,18,22]. There is significant overlap in the aetiology and disease mechanism of hypertension and diabetes [23]. Obesity, inflammation, oxidative stress and insulin resistance are central to the pathophysiological mechanisms for the diseases.

There is a growing concern of the high prevalence of non-communicable diseases such as hypertension, T2DM, obesity, and other cardiovascular diseases in both rural and urban African countries [24,25]. The World Health Organization predicts that Sub-Saharan Africa will experience an increase in the prevalence of cardiovascular diseases over the next decade [26]. This might be a plausible prediction considering the rapid epidemiological and nutritional transitions in Sub-Saharan Africa [27,28].

All the participants were already on antihypertensive treatment. However, the high prevalence of uncontrolled hypertension in the studied population occurred irrespective of the number and classes of hypertensive drugs (Table 2) which strongly suggests clinic inertia. Possible explanation for the observed phenomenon could be due to failure of optimization of doses prior to adding another medication. Hence, combination of two or four drugs did not achieve treatment targets.

Most of the participants were treated with at least three anti-hypertensive medications and thiazide diuretic as the commonest medication, followed by angiotensin converting enzyme inhibitor (perindopril) both of which are beneficial to populations living with concomitant hypertension and diabetes [11,14]. Lack of data on the duration of treatment, dosing schedule and trends of escalation of doses by clinicians in the health facilities limit us from determining the magnitude of clinic inertia and to identify participants with refractory hypertension. Adherence to anti-hypertensive drugs was poor in the studied population which is an important explanatory factor for the overall high prevalence of uncontrolled hypertension. Appropriate interventions in individuals with co-morbid hypertension and diabetes should therefore, focus on enhancing adherence to multiple and concurrent medications. Treating cardiovascular diseases to target levels is imperative and compelling evidences from the literature seem to support this [4–7]. We therefore, hope that the health authorities of Mthatha General Hospital and the district health services will utilize the findings of the study to intervene in order to improve the health outcomes of individuals living with hypertension, diabetes and other cardiovascular diseases.

Despite the scientific successes in diagnostics and antihypertensive drug discoveries, achieving treatment targets remain a daunting task. The present study found high prevalence (75.5%) of uncontrolled hypertension among diabetic patients comparable to studies among hypertensive individuals reported elsewhere [29–35], notwithstanding, the inherent pathophysiological and methodological variations involved in those studies. Beside the improvement in the health outcomes of individuals living with hypertension over a 10-year period in Switzerland reported by Guessous et al. [29], the rate of controlled hypertension never exceeded 40.2% in most countries worldwide. Few hospital survey from rural Ghana, Lupane in Zimbabwe and three regions of Morocco reported similar trend of poor blood pressure control among patients treated for hypertension; 91.1%, 67.6% and 82.8%, respectively [30–32].

As previously reported in the literature [29–35], many sociodemographic and clinical factors may impact on the control of hypertension. Although, there seems to be mixed reports on the association of age and control of blood pressure, better blood pressure control was achieved in older individuals in South Africa almost a decade ago, Kenya and China [33–35]. However, Agyemang et al. [36] reported worse level of blood pressure control among patients 50 years and above in Ghana. Similar trend of poor blood pressure control among younger women was reported in a multi-national study by Nejjari et al. [37]. Our finding of poor control of blood pressure among older men is unique due to the fact that in South Africa particularly among the rural African dwellers, it is observed that older men tend to relocate from cities upon retirement or other conditions of life, to live in the rural communities where access to quality healthcare services are sub-optimal. This age group are often retirees and also, unemployed people, and thus, lack transport money to visit the hospitals. Anecdotally as this may appear, it could not be ignored as a probable explanation for the high prevalence of uncontrolled hypertension in this setting.

It should be noted that men were under-represented in this study and also, being male was associated with higher odds of having uncontrolled hypertension. Few studies have shown a more effective utilization of health facilities by women, which may account for the higher number of women in the study [20,38]. Another probable reason for our finding might be linked to the migratory pattern of young men in this region to the bigger cities in search for jobs. Also, cultural perspectives cannot be excluded as men tend to delay in seeking health care and hence, poor health outcomes. The trend of poor control of hypertension among men (26%) was documented by Steyn et al. [35] in a national survey in South Africa. Also, similar finding of poor control of blood pressure among men was reported in Geneva, Switzerland [29].

Finding of poor control of blood pressure among unemployed individuals might be linked with the rural poorly resourced residence of the participants. Adeniyi et al. [20] highlighted the long distances patients had to travel to keep doctors’ appointments, poor monthly income and absence of doctors at the rural health care facilities which impact directly on the access to monthly reviews, pick up of medications and adherence to lifestyle adjustments required by individuals living with the double burden of hypertension and T2DM. Also, the association of the consumption of western-type diet and excessive alcohol consumption signifies the transitions in nutrition and epidemiological changes experienced by people living in the rural communities. This transitions occur as a result of globalisation which have been reported in many studies in Africa [20,39–41]. Though, alcohol use may affect adherence to chronic medications and thus, poor blood pressure control; we did not profile the behavioural changes related to alcohol in our study and as such, we are cautious to ascribe the poor control of blood pressure in our study to alcohol use.

There was a positive linear association of duration of T2DM and poor blood pressure control (Fig 1). This could be explained by the overall mean of HbA1c was greater than 10% and this relationship was seen with increasing duration of diabetes. Plausible reason for this finding might be related to poor health outcomes of all the chronic diseases in this population. Both the glycaemic control and blood pressure control for the entire population were poor. Hence, health authorities of the district health system of Mthatha need to prioritise primary health care for people living in the rural underserved communities.

Unique trends of characteristics were observed in the studied population; (Table 3); increasing age, monthly income, BMI, T2DM duration and creatinine while decreasing HDL-C, LDL-C, and LDL-C/HDL-C. The association of dyslipidaemia with poor blood pressure control seems complex as both LDL-C and HDL-C tend to decrease across the hypertension groups. The LDL-C levels across the three groups were above the desired level of 1.8mmol/L [11], while HDL-C levels tend to decrease across the groups. The prescription of statins either at therapeutic or prophylactic doses in the population if obtained, would have shed more light on the probable explanation for the trends in lipid levels observed in our study.

The outcomes of care in the study population is worrisome and may be comparable to those individuals who are not yet diagnosed. We may be failing to alter the natural course (prognosis) in the majority of the individuals with concomitant hypertension and T2DM. Hence, the risk of developing both macrovascular and microvascular complications may not have reduced. Our findings in this understudied and poorly resourced area of the country suggest that poor health outcomes of the individuals living with the double burden of non-communicable diseases is factual. Therefore, prioritization of quality and equitable health care service delivery to the rural communities is mandatory in South Africa.

### Limitations

The limitations of the study should be noted. First, it is a cross-sectional study, hence, causality cannot be ascribed to the determinants. The underrepresentation of men in the study did not allow full understanding of the health outcomes in the study population. Therefore, a population-based study is proposed to provide a more robust epidemiological data for the entire population. Lack of information on the dosing regimen of the antihypertensive medications limit us from identifying the proportion of the participants with resistant hypertension. We did not measure urine sodium excretion and could not ascertain the contribution of dietary salt intake in the study population. Notwithstanding these limitations, the findings of the study provide useful epidemiological data; being the first study from this region to highlight the treatment outcomes of individuals living with the concomitant hypertension and T2DM.

## Conclusion

Findings of high prevalence of uncontrolled hypertension in individuals with concomitant type 2 diabetes mellitus in the study highlight the urgency for primary health care re-engineering in the rural communities of South Africa. Male sex, unemployment, excessive alcohol intake, consumption of Western-type diet, aging, clinic inertia, longer duration of T2DM and dyslipidaemia are important determinants of uncontrolled hypertension in the studied population. Treatment to blood pressure targets though feasible in our setting, would require concerted efforts by addressing these determinants and clinic inertia. Overall, a paradigm shift towards integrated management of both infectious and non-communicable diseases is urgently needed to address the health problems in South African population.
